# Seasonal variation of malaria cases in children aged less than 5 years old following weather change in Zomba district, Malawi

**DOI:** 10.1186/s12936-017-1913-x

**Published:** 2017-07-03

**Authors:** Precious L. Hajison, Bonex W. Mwakikunga, Don P. Mathanga, Shingairai A. Feresu

**Affiliations:** 1 Invest in Knowledge, Epidemiology Research Unit, Zomba, Malawi; 2 School of Health Systems and Public Health, Epidemiology & Biostatistics Track, University of Pretoria, 5-10 H.W. Snyman Building, Pretoria, South Africa; 3 DST/CSIR Nanotechnology Innovation Centre, National Centre for Nano-Structured Materials, Council for Scientific and Industrial Research, Pretoria, South Africa; 4 Malaria Alert Centre & Department of Community Health, College of Medicine, University of Malawi, Blantyre, Malawi

**Keywords:** Seasonal variation, Malaria, Outpatient children, Weather change, Malawi

## Abstract

**Background:**

Malaria is seasonal and this may influence the number of children being treated as outpatients in hospitals. The objective of this study was to investigate the degree of seasonality in malaria in lakeshore and highland areas of Zomba district Malawi, and influence of climatic factors on incidence of malaria.

**Methods:**

Secondary data on malaria surveillance numbers and dates of treatment of children <5 years of age (n = 374,246) were extracted from the Zomba health information system for the period 2012–2016, while data on climatic variables from 2012 to 2015 were obtained from meteorological department. STATA version 13 was used to analyse data using non-linear time series correlation test to suggest a predictor model of malaria epidemic over explanatory variable (rainfall, temperature and humidity).

**Results:**

Malaria cases of children <5 years of age in Zomba district accounts for 45% of general morbidity. There was no difference in seasonality of malaria in highland compared to lakeshore in Zomba district. This study also found that an increase in average temperature and relative humidity was associated of malaria incidence in children <5 year of age in Zomba district. On the other hand, the difference of maximum and minimum temperature (diurnal temperature range), had a strong negative association (correlation coefficients of R^2^ = 0.563 [All Zomba] β = −1295.57 95% CI −1683.38 to −907.75 p value <0.001, R^2^ = 0.395 [Zomba Highlands] β = −137.74 95% CI −195.00 to −80.47 p value <0.001 and R^2^ = 0.470 [Zomba Lakeshores] β = −263.05 95% CI −357.47 to −168.63 p value <0.001) with malaria incidence of children <5 year in Zomba district, Malawi.

**Conclusion:**

The diminishing of malaria seasonality, regardless of strong rainfall seasonality, and marginal drop of malaria incidence in Zomba can be explained by weather variation. Implementation of seasonal chemoprevention of malaria in Zomba could be questionable due to reduced seasonality of malaria. The lower diurnal temperature range contributed to high malaria incidence and this must be further investigated.

## Background

Malaria has afflicted people of all ages in the entire past decade and still is one of the major global health problems [1]. Malaria contributes to a significant burden in widespread populations with premature deaths, infirmity from sickness and it inhibits on economic and social development [2]. World Malaria Report 2015, stipulated that, globally, malaria incidence was estimated to be at 214,000,000 infected cases and 438,000 deaths [3]. Malaria was estimated to have contributed to about 82,685 disability-adjusted life years (DALYs), in the year 2010, which accounted to almost 19.6% of all causes of diseases [4]. The disease has remained a major cause of morbidity and mortality in Malawi [5, 6]. Malaria was estimated at 34% of outpatient visitation to hospital in Malawi in 2011. Further in 2015, malaria incidence was estimated at 200 cases per 1000 population.

Malaria is influenced by a lot of environmental factors, which affects its seasonality, distribution, and transmission intensity [7]. It is well known that climate is the significant contributing factor of the spatial and temporal distribution of malaria vectors and pathogens [8, 9]. The major climatic factors influencing malaria transmission are rainfall, temperature and humidity [10, 11].

Temperature and humidity determines the length of mosquito cycle and the sporogonic cycle of the parasite in the mosquito. Parasite transmission and development are regarded to be influenced by climatic conditions especially within the temperature range of 25–30 °C. Parasite development is believed to decrease significantly in the life cycle of mosquito stage when temperature is below 16 °C. Above 32 °C, parasite development slows down considerably hence survival of the vector becomes uncertain. One study assessed the relationship between climate changes and malaria cases in 25 African countries [12]. Reports from this study indicate that as temperature increases above 25 °C and, decreases below 20 °C, malaria cases start to decrease but malaria cases are very stable within 20–25 °C [12]. A similar study also reported that temperature increase can shorten the life cycle of mosquito, hence increases chance of high malaria epidemic [13]. Another study reported that diurnal temperature range variation could also affect development of malaria parasite [14].

Relative humidity of more than 60% is regarded a suitable condition for survival of mosquito [15]. In addition, there is a direct influence of temperature and rainfall on the number and productivity of breeding sites, ultimately the vector density [16]. Monthly rainfall of 80 mm is considered suitable for mosquito life cycle in surface water whereby eggs and larval stages are favorably done. Numerous studies have demonstrated the association between *Anopheles gambiae* complex abundance and rainfall [17]. Malaria sensitivity to climate was demonstrated in 25 African countries, where marginal change in rainfall and temperature, were found to be critical parameters for malaria transmission [12]. High malaria transmission in the tropical highlands in future following climate change effect was predicted to increase [18, 19]. Climate-related epidemics were also reported in Malawi, this was assumed to have been attributed to heavy rainfall following a drought [20].

Under-5-year children are the most susceptible to malaria infections [21]. Malaria was estimated to claim 7.4% lives of between the ages of 6 months and 5 years globally in 2010 [22]. During pregnancy, malaria is estimated to cause different complications ranging from low birth weight to death [23]. Study done by Walldorf et al. [21] reported that school going age children are reservoirs for malaria in Malawi. Additionally, it was reported that malaria accounted for 40% of all hospitalized children of <5 years old. Furthermore, malaria was estimated to cause 40% of deaths in hospitalized people, of which, 50% were <5 years old children in Malawi [2]. Although Malawi is in line with Sustainable Development Goal (SDG) indicator 4.1; (reducing mortality rate of under-5-year children), it has not yet reached the target [24]. According to Malawi SDG end-line survey in 2014, mortality rate for children under-5-years old was at 85 per 1000 live births, which is still high [25].

This study compared how seasonality of malaria and climatic factors reward the number of children <5 years of age from Zomba district, particularly those originating from the lakeshore and highlands.

## Theoretical considerations

Apart from performing correlational analysis between the data types which is important for the determination of how variables (including weather parameters of humidity, rainfall, temperature) may or may not influence other variables in the data (such as malaria cases in lakeshore and highlands), it is also equally useful to perform a time series analysis on data which are collected and recorded in time. The latter analysis helps to understand the history, the present status as well as help to possibly predict future trends. In this work, data on malaria incidence were recorded in time (monthly) between the years of 2012 and 2016 and in many local clinics spread-out geographically within the Zomba district of Malawi. Other data collected in the same time but limited up to 2015 and geographical spaces are the weather conditions.

Of the many models, the Fourier transform has been ubiquitous in the time series analysis for centuries. Its weakness is that it only works with periodic data, i.e. data that have a constant frequency vis-à-vis regarded as stationary time series. Over the years, other methods, such as the wavelet analysis method described by Huang et al. were discovered [26]

1 $$W\left( {a,b;X,\psi } \right) = \left| a \right|^{ - 1/2} \int\limits_{ - \infty }^{\infty } {X\left( t \right)} \;\psi \; * \;\left( {\frac{t - b}{a}} \right)dt$$

In which ψ*(.) is the basic wavelet function that satisfies certain very general conditions for instance our present malaria infection data collected from patients who attended OPD diagnosis. Patients who have been malaria infected but never reported to OPD are not considered in this case; *a* is the dilation factor and *b* is the translation from the origin. 1/*a* gives the frequency scale and b, the temporal location of the event. The physical explanation of Eq. 1 is very simple—that W(a,b;X,ψ) is the “energy” of X of scale *a* at time t = *b*. So this equation can be used to predict the malaria infections at any time t, given the parameters *a* and *b* obtained from the data trend. Morlet wavelet [27] is a subset of Eq. 1 and, in the last two decades, has become popular since it works for data that have gradual frequency changes. The Wigner-Ville distribution, a type of Eq. 1, or sometimes referred to as the Heisenberg wavelet being the Fourier transform popular with electrical engineers [28], but suffers from being similar to the Fourier transform. The evolutionary spectral analysis has been popular in earthquake communities [29] but difficulty in its application lies in the lack of a method to define the basis which is why it is difficult to predict earthquakes up to date.

Other methods, such as the least square estimation of the trend, smoothing by moving averages and differencing to generate stationary data, have been described [30]. Smoothing the data may not be employed in the forecasting the future incidents from the data trends but is useful in making the data less noisy so that forecasting can be plausible. Moving averages and fast Fourier transforms are some of the major smoothing algorithms. Moving averages can be simple (SMA), cumulative (CMA) or exponential (EMA). Simple moving average (SMA) is the unweighted mean of the previous n data. However, here, the mean is normally taken from an equal number of data on either side of the central value [31]. This ensures that the variations in the mean are aligned with the variations in the data rather than being shifted in time. In this case if m_M_, ${\text{m}}_{{{\text{M}}\; - \;1}} , \ldots ,{\text{m}}_{{{\text{M}}\; - \;\left( {{\text{n}}\; - \;1} \right)}}$ are the simple equally weighted mean malaria cases for an n day sample, then

2 $$SMA = \frac{{m_{M} + m_{M\; - \;1} + \cdots + m_{{M - \;\left( {n\; - \;1} \right)}} }}{n} = \frac{1}{n}\sum\limits_{i = 0}^{n\; - \;1} {m_{M\; - \;i} }$$

Similarly, fast Fourier transform filter, which is credited to Cooley and Tukey [32], involves Fourier transforming, for instance the malaria-cases-time data, m(t), into frequency domain, M(f), smoothing M(f) into M’(f) by moving averages and then transforming M’(f) into a new smoothed m’(t) back in time domain. Thus, if m(t) has to be transformed in M(f), we have the forward Fourier transform thus

3 $$M\left( f \right) = \int_{ - \infty }^{ + \infty } {m\left( t \right)\;\left[ {\cos 2\pi ft - j\sin 2\pi ft} \right]dt}$$

And the reverse Fourier transform thus

4 $$m\left( t \right) = \int_{ - \infty }^{ + \infty } {M\left( f \right)\;\left[ {\cos 2\pi ft - j\sin 2\pi ft} \right]df}$$

The process of fast Fourier transform (FFT) filter is thus illustrated in Eq. 4

5 $$m\left( t \right)\;\underrightarrow {FT}\;M\left( f \right)\;\underrightarrow {smooth}\;M^{\prime}\left( f \right)\;\underrightarrow {FT^{ - 1} }\;m^{\prime}\left( t \right)$$

In this paper, smoothing of the time domain malaria cases data is performed by both simple moving average as in Eq. 2 as well as the FFT process as in Eqs. 3 to 5 and then the forecasting of malaria cases by Eq. 1 will be attempted.

It is also worth noting that malaria cases statistics can be heavily dependent on vector populations which in turn are dependent on prevailing weather or environmental conditions and changes. The malaria cases statistics therefore can be modelled as a sensor of the presence or absence of mosquitoes. Dry seasons, when mosquito populations are low, are marked by reduced populations of malaria cases whereas wet seasons show high incidence of the same. For this reason, the population of malaria cases, m(t), can be modelled by the exponential growth during wet season onset and exponential decay in the onset of the dry season. The exponential growth and decay may be simple but may also contain more than two terms due to multivariate nature of the data as follows:

6 $$m\left( t \right) = {\text{ }}\left\{ {\begin{array}{*{20}l} {m_{{w0}} + A_{{w1}} .\exp \left( {t/t_{{w1}} } \right) + A_{{d2}} .\exp \left( {t/t_{{w2}} } \right)\quad wet} \\ {m_{{d0}} + A_{{d1}} .\exp \left( { - t/t_{{d1}} } \right) + A_{{d2}} .\exp \left( { - t/t_{{d2}} } \right)\quad dry} \\ \end{array} } \right.$$

where m_w0_ is lowest number of malaria cases, A_1_ and A_w2_ are the maximum increment in the malaria cases due to change of season, t_w1_ and t_w2_ are the characteristic response times during the wet seasons whereas m_d0_, A_d1_, A_d2_, t_d1_ and t_d2_ have usual meaning for the dry seasons.

Response, S, of the malaria cases statistics to environmental changes and hence to the malaria vector populations is defined as in sensor science [33] to be:

7 $$S = \frac{{A_{1} + A_{2} }}{{m_{0} }}\; = \;\frac{{m_{wet} - m_{dry} }}{{m_{dry} }}$$

## Methods

Existing grouped data on patient numbers and dates of treatment of children <5 years of age (n = 374,246) was extracted from the Zomba health information system for the period 2012–2016. Data on climatic variables were obtained from meteorological department from 2012 up to 2015.

**Fig. 1:**
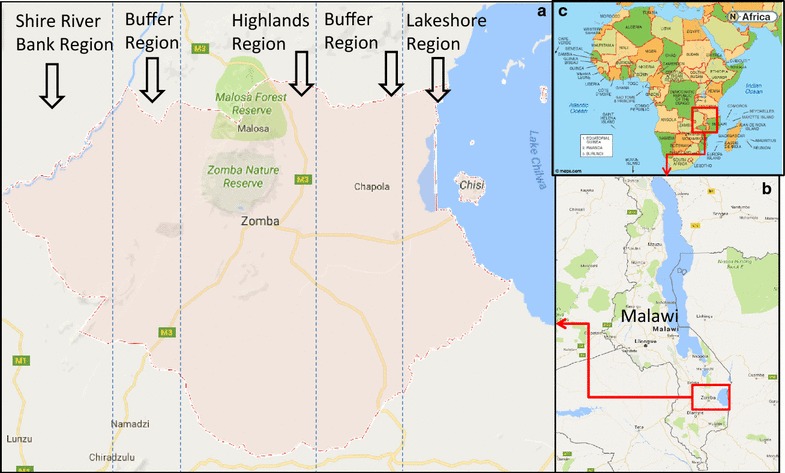
Maps of study area. **a** Zomba district divided into five regions as shown: Shire river bank region, Buffer region, Highlands region, Buffer region and Lakeshore region. **b** Malawi map showing the location of Zomba district and **c** Africa showing the position of Malawi as a country

The Zomba district, consist of Lake Chirwa, Shire River and Zomba Mountain as shown in Fig. 1. The study area was divided into five regions as follows: (1) Shire river bank region, (2) Buffer region between Shire riverbank region and the Highlands region, (3) the Highlands region (4) Buffer region between Highlands region and Lakeshore and (5) Lakeshore region. Additionally, Zomba district has one central hospital, and one rural hospital, recently upgraded. All the patients admitted to these two hospitals come from either health centres or clinics. All the surveillances for all patients are reported in the outpatient department first, before any admission. Due to this, all patients are first regarded and reported as outpatients before recorded as admissions for those that end up being admitted. Zomba district health surveillance is solely dependent on 17 health centres and 3 public clinics. Furthermore, Zomba district gets its data from 10 Christian Health Association of Malawi (CHAM) hospitals and 3 clinics, one for Malawi Army and 2 for Malawi Police. According to demarcation of regions in this study, Zomba district accounted for 7 health facilities in lakeshore region and 6 health facilities in the highlands. The rest of health facilities were neither part of highland or lakeshore, but are in the buffer region. Data for the total population of under-5-year children in the respective catchment areas of each health facility were extracted from Zomba district health information system.

The study only used confirmed malaria cases data by microscopy and largely with malaria Rapid Diagnostic Test (RDTs), though it is also reported that RDTs can also fail to detect low density parasitaemia (<200 parasites/µl) [34]. Monthly total for general out patient department (OPD) visits, monthly total malaria cases, monthly total malaria deaths, monthly total general deaths, and total population of under-5-year children in respective health facility catchment areas for Zomba district were collected from the HMIS. Data were extracted electronically from the Health Management Information System (HMIS). For incomplete electronic data, manual verification with the register at the health facility was done. The manual verification was initiated when the electronic data showed a drastic drop or increase of the malaria cases, e.g. the following month drops from 1000 cases to 0 cases. In case there was a difference between register records and electronic data, register data were regarded as correct. Monthly totals of humidity, temperature and rainfall were extracted from the meteorological department.

Apart from correlating weather data to malaria cases data and also apart from time series analysis of the time-domain malaria cases data, this study adopted an idea on how to calculate seasonality from sensors science by Mwakikunga et al. [33]. The study considered two seasons of malaria epidemic in Malawi. November to April was deemed rainy season while May to October dry season. Rainy season was represented as season (a), dry season as (b). To calculate seasonality the formula below (similar to Eq. 7) was used.

8 $$S_{wd} = \frac{{m_{wet} - m_{dry} }}{{m_{dry} }}\; \times \;100\%$$

where S_wd_ is the seasonality between wet and dry seasons, M_wet_ and M_dry_ are the malaria infected population during the wet and dry season respectively. This implies that the bigger the percentage the higher the seasonality.

The study calculated the cumulative incidence and morbidity rate per 100 of children aged <5 years in Zomba for the period of 2012–2015. Incidence, I, was defined as *I* = *m*/*P*_T_ where m is the number of malaria cases in a given area and *P*_T_ is the total population in a given area. Morbidity rate, M_r_, was defined as Mr = *im*/*P*_T_ where *im* is number of malaria infected children in a given area. The study then compared the malaria incidence rate and morbidity rate between the highland region and lakeshore region and came up with the cumulative trend of monthly malaria cases [35].

Two variables were generated from weather parameters of mean, maximum and minimum temperature and relative humidity readings for a given area. One called diurnal temp range defined as *T*_max_ − *T*_min_, and the other named diurnal humidity range [*RH*_max_ − *RH*_min_] [36].

Lastly, the incidence rate and percentage of peak malaria in 4 consecutive months were calculated as the total incidence for the 4 peak consecutive months over the total population for under-five children in the area; and the total incidence for the 4 peak consecutive months over the total malaria incidence for the whole year, respectively. These calculations were stratified according to geographical location, i.e. the whole district, lakeshore and the highland.

## Results

Each variable used in the study is presented in the statistical summary in Table 1. The period of time of observation for each variable in this table was 57 calendar months. The variables included are malaria cases according to geographical locations, general infections including malaria cases and climatic conditions.

**Table 1:** Statistical summary table for malaria and climatic variables from Zomba district within 2012–2016

Variable	Definition of variable	Observation in calendar months	Mean # of cases	Std. dev	Min # of cases	Max # of cases
Malaria <5 Zomba	Total malaria case for Zomba district	57	13,131.44	48,771.69	2285	374,246
Zomba highlands <5 malaria	Total malaria case for highlands in Zomba	57	1661.68	6179.78	250	47,358
Zomba lakeshore <5 malaria	Total malaria case for lakeshore in Zomba	57	2752.84	10,228.22	38	78,456
Zomba total morbidity for <5	General morbidity for <5 in Zomba	36	14,149.74	1094.74	8384	25,043
Rainfall	Rainfall readings for Zomba	36	3.66	5.35	0	20.3
Mean temp	Average temperature for Zomba	36	22.33	2.45	17.75	26.85
Diurnal temp range	Minimum and maximum difference of temperature for Zomba	36	10.26	1.70	6.3	12.5
Mean humid	Average humidity for Zomba	36	61.28	9.83	45.1	77.9
Diurnal humid range	Minimum and maximum difference of humidity for Zomba	36	17.35	5.77	7	27.8

**Fig. 2:**
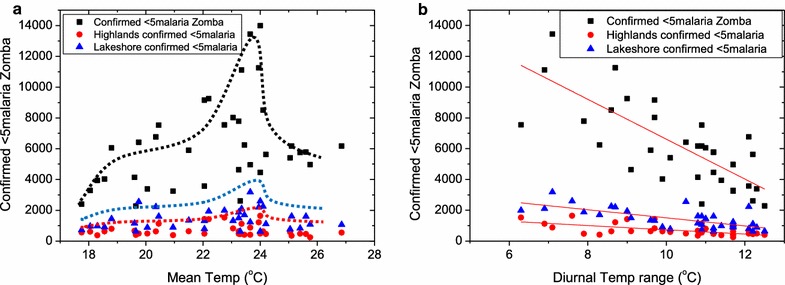
Correlation between mean temperature (**a**) diurnal temperature ranges to malaria (**b**) and general under-five morbidity in Zomba district, Malawi. **a** There is a general increase in malaria and morbidity cases as mean temperature increases. This increase reaches a peak at 24 °C where the number of malaria and morbidity cases drastically drop. A Gaussian fit which is indicated by a *solid trend line* confirms this critical mean temperature of 24 °C. **b** Suggests that rapid and large changes in diurnal temperatures lead to lower reported malaria cases, a finding which leads to the suspicion that vector breeding is curtailed by these temperature variations

**Fig. 3:**
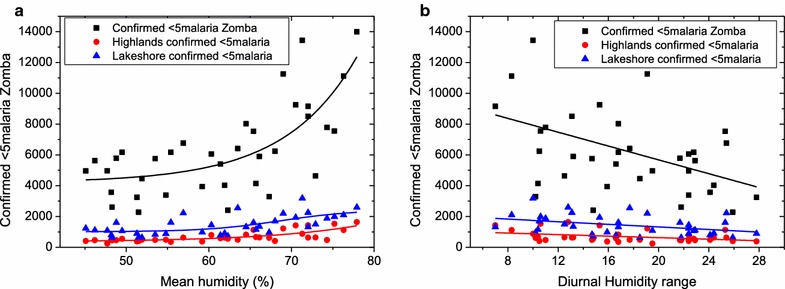
Malaria cases among under-5-year of age children plotted against (**a**) relative humidity prevailing in that particular month and (**b**) average diurnal humidity range. The data suggest that humidity level below 40% and above 80% lead to reduced, if not zero malaria cases. Also the rapid changes in humidity lead to less infections possibly owing to vector population reduction under large fluctuations in humidity

Malaria can be correlated to mean temperature (Fig. 2a) and diurnal temperature changes (Fig. 2b) as well as mean relative humidity (Fig. 3a) and changes in relative humidity (Fig. 3b). In Fig. 2a, the rising trend of malaria cases as mean temperature increases is clearly observed. The malaria cases reach their peak at a critical temperature of 24 °C beyond which numbers reported start to diminish. It must be noted that no malaria cases are observed at temperature lower than 16 °C and greater than 27 °C. In Fig. 2b, it is observed that diurnal variations in the temperature affect malaria cases negatively (r = −1295.57 95% CI −1683.38 to −907.75 p value <0.001). Large diurnal temperatures lead to lower infections. This suggests that large temperature variations lead to less vector breeding rates and hence less vector populations.

In Fig. 3a, increasing relative humidity also proportionately leads to increased number of under-five malaria cases reported. No malaria cases are reported at relative humidity levels of lower than 42% and any above 78%. Similar to diurnal temperatures, in Fig. 3b, one observes that increasing diurnal humidity reduces the reported under-five malaria cases (correlation coefficient, R^2^ = 0.176 [All Zomba], r = −224.45 95% CI −381.10 to −67.79 p value <0.006, R^2^ = 0.130 [Zomba highlands] r = −24.85 95% CI −45.06 to −4.65 p value <0.017 and R^2^ = 0.119 [Zomba Lakeshores]) (r = −42.13 95% CI −77.95 to −6.30 p value <0.023). Again this could be attributed to harsh conditions imposed to vector breeding when there are large variations in humidity.

**Fig. 4:**
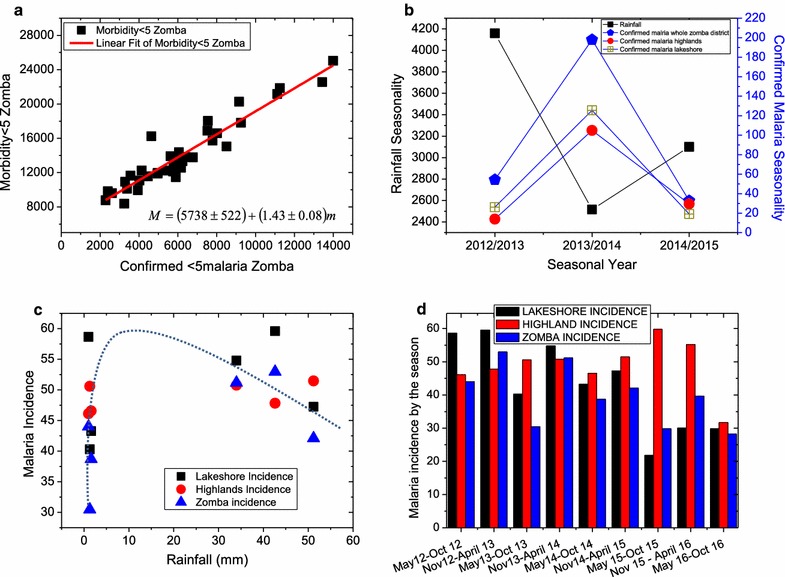
Are presented charts on general morbidity compared to malaria cases (**a**), seasonality of rainfall and that of malaria plotted versus seasonal year on the same chart (**b**), incidence of malaria in a given area against rainfall (**c**), and incidence of malaria against season from 2012 to 2016 (**d**). **a** A positive linear correlation (R^2^ = 0.89899) between the total morbidity plotted against total malaria infected cases, **b** malaria cases seasonality regardless of location scales inversely as rainfall seasonality, **c** incidence of malaria in different locations of Zomba district plotted versus rainfall levels showing a critical level of about 8 mm of rainfall that leads to maximum malaria incidence of more than 60 out of 100 children and **d** incidence of malaria in different areas plotted against season from 2012 to 2016 showing a general decline in malaria prevalence in this period

The effect of malaria cases on the total morbidity of the population can be measured by plotting the morbidity data against malaria cases data. This kind of plot is illustrated in Fig. 4a and this paper presents a linear scatter plot to show the correlation of general morbidity versus malaria morbidity for children aged <5 year in Zomba district. Simple linear regression was fitted in order to determine the relationship of general morbidity for children aged <5 years and malaria morbidity for children aged <5 years in the Zomba district (β = 0.67 95% CI 0.595 to 0.750 p value <0.001). It can be observed in Fig. 4a that there is a strong positive and linear correlation (R^2^ = 0.89899) between total morbidity and total malaria infected under-five children. The linear fit reveals an intercept of (5728 ± 522). This means that, in the absence of malaria cases, there were between 5126 and 6260 infections among under-five children from 2012 to 2015 owing to other diseases such as HIV-AIDS, tuberculosis, diarrhea, upper respiratory tract infections and malnutrition. This means an average of about 1000 sicknesses per year owing to other diseases apart from malaria. At a slope of 1.43 ± 0.08, the graph in Fig. 4a suggests that for every 143 (between 135 and 151) under-five children infections, 100 (between 95 and 109) infections are attributed to malaria. This shows how important malaria cases are to the total morbidity burden in Zomba district. This finding could be generalized to other parts of Malawi, Africa and other parts of tropics around the world.

Figure 4b shows the trend of the seasonality of rainfall and malaria cases with seasons. This graph shows that each time rainfall seasonality reach a minimum, malaria cases reach a peak and vice versa. This conclusion is only half of the truth as is shown in Fig. 4c where incidence of malaria in all areas in Zomba has been plotted against rainfall. The latter figure shows that malaria incidence is about 30 out of 100 when there is no rainfall, but it rises sharply up to some critical rainfall value of about 8–15 mm beyond which the malaria incidence decrease at a slower rate than the rate at which it had risen before. The rainfall-dependent decline in malaria incidence could be attributed to the decrease in vector (mosquito) population during heavy rain conditions which is further attributed to poor mosquito breeding conditions when there are fast-moving waters and less still waters, pools and puddles.

Figure 4d is a plot of malaria incidence data for highlands, lakeshore and the whole Zomba district. Malaria incidence in children aged <5 years in Zomba district accounts for 56.46%. The incidence of malaria in all locations shows a decreasing trend in time (season) from 2012/13 to the end of the data collection season in 2016. The lowest incidence of 22 out 100 children was recorded in the lakeshores in the season between May 2015 and October 2015.

This study further calculated cumulative malaria incidence and malaria morbidity rate in Zomba district, for a 4 year time period (2012–2015 morbidity rate and 2012–2016 for incidence rate). The incidence for the entire Zomba district, was 57 per 100 children aged <5 years. The highland incidence was 79 per 100 while, Zomba’s lakeshore was 57 per 100. Malaria morbidity rate, for Zomba district was estimated at 44.33%, lakeshore 55.14% and highlands 58.7%. During this period, Zomba district had high incidence of malaria throughout the year in all seasons at all locations (see Fig. 4d). Seasonality of malaria in Zomba district was similar in highland and lakeshore areas.

**Fig. 5:**
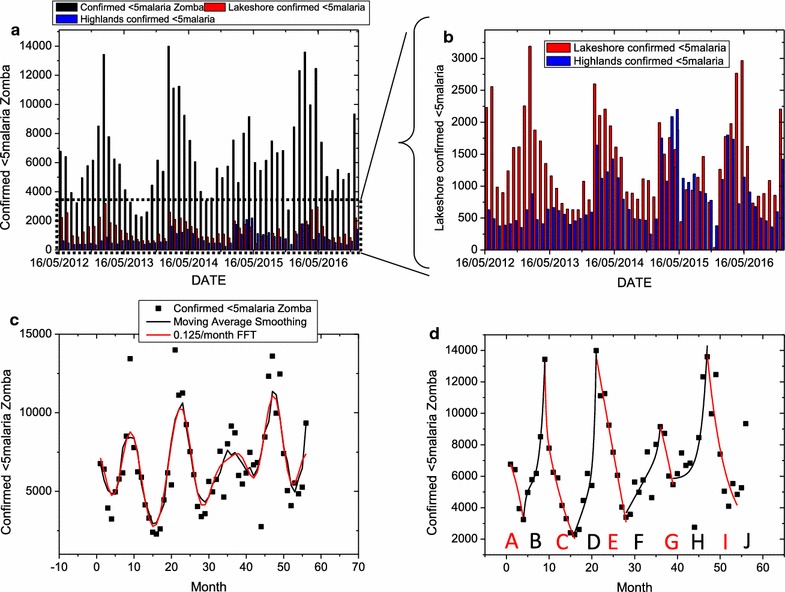
**a** Time series plot between the years 2012–2016 of under-five malaria cases in the highlands and lakeshores and the total number in Zomba, **b** a magnified time plot of the under-five malaria cases in the highlands and lakeshore areas only, **c** under-five malaria cases data fitted with smoothing moving average and low pass FFT filter and **d** the same data fitted with exponential growth and exponential decay curves. Note that in both highlands and lakeshore, the number of malaria case rises during the wet seasons and decrease during the dry until in the year 2014 when this pattern becomes distorted. Also regardless of whether rainfall goes down to zero or not, the number of malaria cases in both lakeshores and highlands is non-zero. Also note that the lakeshore areas account for consistently more under-five malaria infections all the time than the highlands. The fitting of the moving average and FFT filter equations reveals a frequency in the data pattern of malaria cases rising or falling at a rate of 0.125 per month (or a periodicity of 8 days). The fitting of the exponential growth and decay also leads to response times as low as 4.5 months (about 135 days) per malaria season, *A*, *B*, *C*, *D*, *E*, *F*, *G*, *H*, *I* and *J* as indicated in **d**

In Figs. 4d and 5, it can be noted that, in both highlands and lakeshore, the number of malaria cases rose during the wet seasons and decreased during the dry seasons until in the years between 2013 and 2014 when this pattern became distorted. Also regardless of whether rainfall went down to zero or not, the number of malaria cases in both lakeshores and highlands was non-zero. Also note that the lakeshore areas accounted for consistently more under-five malaria infections all the time than the highlands. In all the panels of Fig. 5, the general trend is that the under-five malaria infected populations are oscillating but at a decreasing rate from 2012 up to 2015 and started slightly rising again in 2016. This decreasing trend in the reported malaria cases could be due to increasing use of mosquito nets, general improvement in the malaria awareness by the population of Zomba, owing to the massive campaigns in malaria education by several organizations. This could also be attributed to changes of climatic conditions.

The fitting of the moving average and FFT filter equations (Fig. 5c, d) reveals a frequency in the data pattern of malaria cases rising or falling at a rate of 0.125 per month (or a periodicity of 8 days). The fitting of the exponential growth and decay also leads to response times as low as 4.5 months (about 135 days) per malaria season, A, B, C, D, E, F, G, H, I and J as indicated in (d). The parameters of A_1_, A_2_, t_1_, t_2_ and y_0_ are summarized in Table 2. It can be noted from Table 2 that the response times range from a few days (about 12 days) to as high as 6 months as the years progress from 2012 to 2016. In contrast the values A_1_ and A_2_, which signify changes in the number of malaria cases reported above the minimum A_0_, initially increased from 2012 up to 2014 after which these maximum changes dropped as the years wore onto 2015.

**Table 2:** Summary of parameters of A_1_, A_2_, t_1_, t_2_ and y_0_ from Eq. 6 fitted onto data for under-five children reported to be infected with malaria in Zomba between 2012 and 2016

Season	y0	A1	t1	A2	t2
A	10536.23	−1510.54	−4.5414	−1510.54	−4.5414
B	5159.53	1.74	1.06	5159.53	−0.57
C	−2643.81	1.32E+19	0.25	39507.41	7.4865
D	−7491.52	3214.893	14.17922	6.40E−16	0.47869
E	−185,430	117265.1	128.1759	117265.1	128.1773
F	−194,118	6.17E−24	0.592	184550.1	404.3063
G	7128.465	−4.72E−11	−0.93846	4.72E−11	−0.93846
H	5817.121	9.36E−11	1.49484	9.46E−11	1.49482
I	1828.639	3.40E+08	4.29071	3.40E+08	4.29042
J	22300.28	−337.493	−12.1242	−337.493	−12.1242

**Fig. 6:**
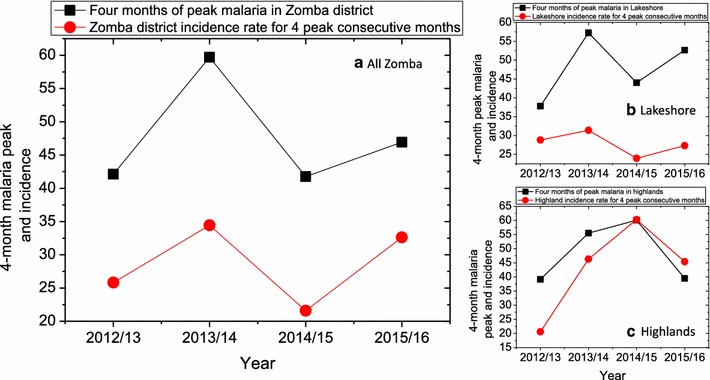
**a** Four consecutive months of peak malaria percentage and incidence rate of all Zomba, Zomba Lakeshore **b** and Zomba highlands **c** for the period from 2012 to 2016

Figure 6 presents the calculated percentages and incidence rate over the years from 2012 to 2016 for 4 consecutive months of peak malaria. This was to ascertain whether Zomba qualifies for seasonal malaria chemoprevention. From the figure, in terms of the incidence rate limit of 10% minimum, the results suggest that Zomba qualifies to implement SMC but because the percentage of malaria cases does not exceed 60% in all the years then Zomba does not qualify. Since for an area to qualify for SMC, it needs to satisfy both criteria, therefore the study may conclude that Zomba does not qualify for SMC.

## Discussion

There is a number of studies supporting the findings that mean temperature and humidity is largely associated with malaria spread. In agreement to this, a study done by Kazembe et al. in northern of Malawi, found that temperature and humidity affects mosquito breeding and survival [37]. This study found that, malaria spread is at peak when temperature is at 24 °C according to Fig. 2a results and, in disagreement to this, a study done by Kazembe et al. where they reported that in Malawi, the favourable temperature for malaria transmission ranges 27–32 °C [38]. This could be the behaviour shifting of mosquitoes in regards to breeding and favorite condition for malaria parasite transmission. These findings correspond to the finding of this study which reported that there was linear correlation between malaria transmission to average temperature in Fig. 2a and relative humidity in Fig. 3a in Zomba district. A small difference between minimum and maximum temperature in Zomba, for the 4 years of the data collected for the study in Fig. 2b, had led to high malaria incidence.

At the same time this study finds that if the difference is lower than 6 °C and, greater than 13 °C there was no malaria present. In 4 years of data collection, minimum and maximum temperature fluctuation has been in converging manner in Zomba district, hence malaria transmission decreased. Although it is well known that mosquito transmit malaria, this does not explain why malaria is negatively affected by diurnal temperature range? This needs further investigation in order to pin down this type of correlation.

This study finds that 67% of variation in general morbidity could be explained by malaria cases in Zomba district according to Fig. 4a. This indicates that, malaria still plays a bigger role on the disease burden in Malawi. These findings are also in agreement with another study that reported why there is no decline in malaria in Malawi [5]. Understanding this variability, could make a bigger difference in terms of resources management for health services in Malawi.

Generally, the seasonality of malaria trend decreased in 2012 through 2015 hence, malaria transmission was observed all year round. In all locations, including highland and lakeshore, seasonality of malaria, increased in every alternate year and decreased in the same manner.

Figure 4b shows that when the percentage of malaria seasonality increased in 1 year the following year the percentage decreased. Although rainfall had similar trend pattern, with malaria transmission, throughout the years of study in all the locations, this study showed that rainfall seasonality was very high compared to malaria transmission. The seasonality of rainfall started to decrease after 2014 which agrees with the general declining trend in malaria cases from these years in Fig. 4c. The general decline in malaria incidence could be due to the falling rainfall but could be also due to increasing awareness by the Zomba population on the use of mosquito nets and malaria control. Additionally, there was no significant drop in malaria transmission during the dry season compared to the wet season. Malaria epidemics were oppositely linked to seasonality of rainfall hence supporting the hypothesis that, malaria transmission occurs all year round and is largely associated with temperature and humidity in Zomba district of Malawi.

Malaria was still a major disease burden in Zomba district of Malawi within 5 years (2012–2016). The incidence for children <5 year of age for the whole district was 56.46% while the highest incidence was observed in highlands about 78.93 and 56.68% in the lakeshore areas. These findings, contradicts findings from other studies, which indicated that malaria burden is higher in lakeshore areas compared to highlands areas. This could be because of weather change within the study period. Zomba district was hotter in these periods, which could suggest that even in the highlands, provided conducive environment for mosquito breeding hence increases malaria transmission.

It can be further noted in Fig. 4d that between May 2012 and April 2013, also from November 2013 to April 2014, malaria incidence in the lakeshores was higher than in the highlands. The opposite is true for the rest of the periods until 2016. One could suggest several factors including, economic status of the households or malaria prevention campaigns which were being rolled out in the district during these periods. Additional to that, this could be because Zomba has two CHAM health facilities in the highland areas, where patients pay for the services. Therefore, it can be assumed that other people who are not critically ill may choose not to go to hospital instead decide to take over the counter drugs. It is possible that patients may prefer to go a long distance to the public hospital outside their study demarcated region hence their surveillance details be recorded in different study region which could bring bias. This might lead to most public health facilities reporting more malaria cases than private health facilities.

This study noted also that the time series analysis of the malaria in highlands and lakeshores suggest that, malaria cases are sympathetic to rainfall. However, this trend gets distorted from 2014. This study suspects that there could be weather changes that disturb the mosquito breeding trends. In all the panels of Fig. 5, the general trend is that the under-five malaria infected populations are oscillating but at a decreasing rate as can be seen from 2012 up to 2016. This decreasing trend in the reported malaria cases could be due to increasing use of mosquito nets, general improvement in the malaria awareness by the population of Zomba owing to the massive campaigns in malaria education by several organizations. This could also be attributed to changes on climatic conditions.

With the parameter obtained from time series analysis in Fig. 5, it is possible to predict by way of the inverse of the wavelet analysis in Eq. 1 that the under-five malaria infections in Zomba will decline in the coming years after 2016 and that this decline will keep oscillating at a period between 8 days (or rate of 0.125 per month) to 6 months (or rate of 0.167 per month) depending on the prevailing climatic and social economic conditions. The changes in infected population will increase and decrease at the rate of about 150,000 infections per month on average in the coming years.

## Conclusion

This study concludes that, climate and weather fluctuations are associated with the diminishing of malaria seasonality regardless of strong seasonality of rainfall, in Zomba district. Referring to Fig. 6, the percentage of malaria in the consecutive 4 months of peak malaria in all location does not qualify for seasonal malaria chemoprevention therapy which is constantly below 60%, almost in all the years. Though on other hand, the incidence rate of malaria in 4 consecutive months of peak malaria cases was always higher than 10%, which qualifies Zomba for SMC. Nevertheless, this study failed to show enough evidence that Zomba district qualifies for SMC, hence do not recommend implementation of seasonal malaria chemoprevention therapy in Zomba district. The time series analysis of the data for under-five malaria infected cases show that the numbers in highlands are consistently lower than in the lakeshores, that all data fluctuate with season—increasing during wet seasons and decreasing during dry season—and that there is a general decreasing trend of numbers of infected under-fives from 2012 to 2016. The lower different ranges in diurnal temperatures contributed to high malaria prevalence, but this must be further investigated.

## Abbreviations

CHAM: Christian Health Association of Malawi
DALY: disability-adjusted life years
HMIS: health management information system
MDG: millennium development goal
MRDT: malaria rapid diagnostic test
OPD: out patient department
